# Nutrient Patterns and Their Association with Socio-Demographic, Lifestyle Factors and Obesity Risk in Rural South African Adolescents

**DOI:** 10.3390/nu7053464

**Published:** 2015-05-12

**Authors:** Pedro T. Pisa, Titilola M. Pedro, Kathleen Kahn, Stephen M. Tollman, John M. Pettifor, Shane A. Norris

**Affiliations:** 1 MRC/Wits Developmental Pathways for Health Research Unit, Department of Paediatrics, Faculty of Health Sciences, University of the Witwatersrand, Johannesburg 2193 South Africa; E-Mails: titilolapedro@gmail.com (T.M.P.); John.Pettifor@wits.ac.za (J.M.P.); Shane.Norris@wits.ac.za (S.A.N.); 2 MRC/Wits Rural Public Health and Health Transitions Research Unit (Agincourt), School of Public Health, Faculty of Health Sciences, University of the Witwatersrand, Johannesburg 2193, South Africa; E-Mails: Kathleen.Kahn@wits.ac.za (K.K.); Stephen.Tollman@wits.ac.za (S.-M.T.); 3 Centre for Global Health Research, Umeå University, Umeå SE-901 87, Sweden; 4 INDEPTH Network: Network of Demographic Surveillance Sites-www.indepth-network.org, Accra, Ghana

**Keywords:** nutrient patterns, adolescents, rural, South Africa, transition, Agincourt health and demographic surveillance system

## Abstract

The aim of this study was to identify and describe the diversity of nutrient patterns and how they associate with socio-demographic and lifestyle factors including body mass index in rural black South African adolescents. Nutrient patterns were identified from quantified food frequency questionnaires (QFFQ) in 388 rural South African adolescents between the ages of 11–15 years from the Agincourt Health and Socio-demographic Surveillance System (AHDSS). Principle Component Analysis (PCA) was applied to 25 nutrients derived from QFFQs. Multiple linear regression and partial R^2^ models were fitted and computed respectively for each of the retained principal component (PC) scores on socio-demographic and lifestyle characteristics including body mass index (BMI) for age Z scores. Four nutrient patterns explaining 79% of the total variance were identified: PCI (26%) was characterized by animal derived nutrients; PC2 (21%) by vitamins, fibre and vegetable oil nutrients; PC3 (19%) by both animal and plant derived nutrients (mixed diet driven nutrients); and PC4 (13%) by starch and folate. A positive and significant association was observed with BMI for age Z scores per 1 standard deviation (SD) increase in PC1 (0.13 (0.02; 0.24); *p* = 0.02) and PC4 (0.10 (−0.01; 0.21); *p* = 0.05) scores only. We confirmed variability in nutrient patterns that were significantly associated with various lifestyle factors including obesity.

## 1. Introduction

The assessment of food and/or nutrient patterns and their relation with non-communicable diseases (NCDs) and obesity is an alternative to the traditional approach focusing on single foods or nutrients. The traditional approach is limited in its ability to demonstrate the impact of nutrient intakes on NCD outcomes because of difficulties in explaining interactions between nutrients and in the lack of ability to detect small effects from single nutrients [1]. Identifying food or nutrient patterns is less complex methodologically and more relevant from a biological and physiological point of view as they allow the analysis of a small number of patterns rather than an array of individual foods and intakes of nutrients that are usually inter-correlated [2,3]. Thus this approach offers a strong complementary strategy to capture the intrinsic complexity of diet, the inter-relationships between different components and the heterogeneity in food and nutrient patterns existing within and between populations [1,4].

Both food and nutrient pattern analyses have been conducted on usual food consumption derived from quantitative food frequency questionnaires using exploratory dimension reduction methods (*i.e.*, principal component analysis) to empirically derive patterns. These multivariate approaches aim to summarize a large number of correlated dietary variables (foods, food groups, nutrients or biomarkers) into fewer independent components (the so called “patterns”) explaining most of the dietary variability despite large within and between variations [1,5,6,7]. In contrast to food patterns, limited work has been done on nutrient patterns analysis to date, [3,8,9,10,11,12,13,14,15,16,17,18,19,20,21,22] with no data available for either approach in Africa.

South Africa, a country undergoing a rapid health transition, characterized by a triple burden of disease including infectious-related under-nutrition illnesses, HIV/AIDs and tuberculosis, and emerging NCDs [23,24,25], has evidence suggesting that the black rural South African population, who were once protected in terms of chronic diseases and obesity, are increasingly susceptible [24]. This could in part be attributed to shifting from traditional prudent diets to high energy, high fat diets, to increasing exposure to less-nutrient-dense foods, and to increasing sedentary behaviour [23,25,26,27]. Such evidence in African adolescence is scarce and deserves further attention. The few data available suggest increases of childhood and adolescent obesity levels [28,29,30,31]. Adolescence, a critical phase characterized by increased vulnerability and exposure to inappropriate diets, could be a major determinant of obesity or developing NCDs in later adulthood [32,33]. To date, no information is available in Africa, characterizing either food or nutrient patterns and associating them with various outcomes including obesity. In this paper, we aim to identify and describe the diversity of nutrient patterns and how depicted patterns associate with socio-demographic and lifestyle factors including body mass index (BMI) in rural black South African adolescents.

## 2. Materials and Methods

### 2.1. Study Population and Design

This cross-sectional study was nested within the Agincourt Health and Socio-demographic Surveillance System (AHDSS), which has been described in detail previously [34,35]. Participants were recruited in 2009 and a sub-sample of 600 participants between the ages of 7–15 years were randomly selected from 3511 children and adolescents who had participated in a 2007 growth survey in the Agincourt sub-district of Mpumalanga Province in South Africa [31]. The original 2007growth study randomly selected children and adolescents between the ages of 1 and 20 years (~100 boys and 100 girls for each year of age) who had lived in the study area at least 80% of the time since birth, or since 1992 when enrolment into the Agincourt HDSS had begun. A random sample of children was drawn from each age-sex-village stratum in proportion to the population size of the village. For this present analysis 388 participants aged 11–15 years (Boys: *N* = 193; mean age 13.53; Girls: *N* = 195; mean age 13.60) on whom dietary data were collected, were included. To ensure that this sub-sample was representative of the larger 2007 study sample [36] we compared various socio-economic status (SES) parameters between the samples, and found no differences (data not shown). Comprehensive details of the methods of recruitment and design have been published elsewhere [34,35].

### 2.2. Ethical Approval

Ethical approval was granted by the University of the Witwatersrand Committee for Research on Human Subjects (Ethics number: M090212), and from the Mpumalanga Provincial Government’s Department of Health. Parental consent and participant assent were secured after full explanation of the study objectives and testing procedures.

### 2.3. Measurement of Diet, Lifestyle Factors, Anthropometric Indicators and Socio-Demographic Information

Diet: Usual diet was assessed for each adolescent using an interviewer-administered quantitative food frequency questionnaire (QFFQ) developed for use in South Africa (SA) [37]. The interview took on average 40 minutes to complete and the QFFQ includes a total of 214 commonly eaten foods [37]. Analyses of 11 dietary surveys conducted in rural and urban SA since 1983 were used to derive these food items, and the list includes all foods eaten by at least 3% of the population [38]. To cater for illiteracy and to improve recall ability, this QFFQ utilizes food flash cards (high quality photographs) of all the food items [39].

Data were collected on the previous week’s (7 day) dietary intake, including convenience food products, in order to estimate habitual intake for each participant. Participants were asked to separate the food flash cards into a series of piles: firstly, they went through each food card and created a pile of food items they ‘rarely/never’ ate or drank. Thereafter, the remaining food cards were divided into a pile of food items they eat/drink less frequently (‘occasional’), and a pile they eat regularly and in the past seven days. The participant was then prompted for information on the frequency and amounts of the regular food items in their diet consumed, the details of which were recorded on the QFFQ [37].

Portion sizes were estimated using household measures and a combination of two-dimensional life-size drawings of foods and utensils, and three-dimensional food models as described and validated by Steyn *et al.* [40]. Items eaten occasionally or rarely/never were also recorded. Coding involved the conversion of the household measures (for example one cup/one serving spoon/one slice) to grams so that an average intake over the previous seven days could be calculated. The quantity and frequency of all consumed foods were recorded and expressed in g/day. Nutrient composition of foods was calculated and all conversions were based on the South African food composition tables [41].

Anthropometry: Height (in mm) was measured using a stadiometer (Holtain, UK) and converted to metres (m), and weight was measured to the nearest 0.1 kg using an electronic bathroom scale. All participants were measured wearing light clothing and without shoes. BMI was calculated as weight in kilograms (kg) divided by height (m)^2^. BMI for age Z-scores were generated using WHO 2007 growth reference standards [42] for children aged 5 to 19 years and obesity is defined as a score above 2SD. Waist circumference was measured using an inelastic tape measure midway between the tenth rib and the iliac crest. Hip circumference was measured at the level of maximum width of the buttocks with the participant standing. Waist-to-hip ratio was calculated by dividing waist (cm) by hip (cm) circumference and waist-to-height ratio was calculated by dividing waist circumference (cm) by height (cm).

Pubertal staging: Pubertal staging was assessed using the Tanner 5-point pubertal self-rating scale which has been validated previously for black South Africans [43]. Genital development in boys and breast development in girls were used to define pubertal stages. Participants were classified as early-puberty (≤Tanner stage 2), and mid-puberty (Tanner stage > 2) [43].

Physical activity: A questionnaire quantifying total physical activity (PA) for the previous 12 months was administered via interview. The questionnaire was developed to be appropriate for South African children, and has been used [44] and validated on urban South African children [45]. Reported frequency and duration of all physical activities (physical education, extra-mural school and club sport, informal physical activity, and walking to and from school) and sedentary activities were recorded. The most reliable (and most complete data) proxy for attaining one’s physical activity level was the total time in minutes spent walking to and from school per week [46] and this parameter was used as a covariate in the present analysis.

Socio-demographics: A variety of socio-demographic and other related data were included from the growth survey conducted two years previously. These included data on the participants’ mothers (age, education level, marital/union status and whether she resides with the participant or not) and SES. These variables and others have been described in detail previously [31].

### 2.4. Data Analysis

Data were analyzed using SPSS statistics software version 20. Principal Component Analysis (PCA) was used to depict nutrient patterns [47] based on the QFFQ derived intake of 25 nutrients. Total fat was divided into monounsaturated, polyunsaturated, saturated fatty acids and cholesterol, whilst total available carbohydrates were divided into starch and sugars (monosaccharides and disaccharides). Total proteins were additionally divided into animal and plant proteins. Alcohol consumption was considered as a main lifestyle factor and was not included in the list of variables to derive nutrient patterns. Additionally alcohol intakes in this adolescent population were negligible 0.02 (+/−0.28) g day^−1^).

Variables were log transformed (natural log) after comparing various analysis options with regard to proportion of variance captured. Log transformation provides an advantage as it renders the variances and covariances independent of scale. PCA was applied with the covariance matrix, rather than the correlation matrix. Variance was based on rotated sums of squared loadings and the Varimax with Kaiser Normalization was used as the orthogonal rotation method, as it maximizes the loading of each variable on one of the extracted factors whilst minimizing the loading on all other factors. In order to capture variability of nutrient intakes independently from variation in energy intake, nutrients (log variables) were adjusted for log total energy intake when applying PCA using the multivariate (standard) method [48]. PCA were conducted on both sexes combined and separated. As comparable patterns were observed in both sexes in PCA the final results are presented for both sexes combined.

**Figure 1:**
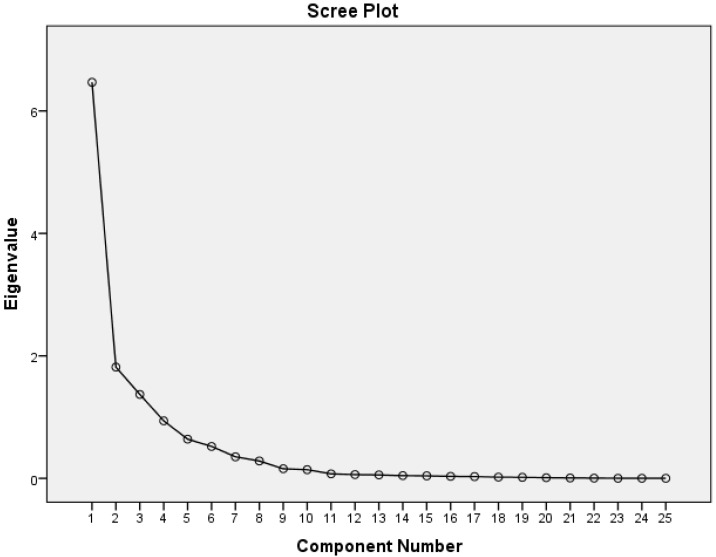
Scree plot of Eigen values after Principal Components Analysis.

**Figure 2:**
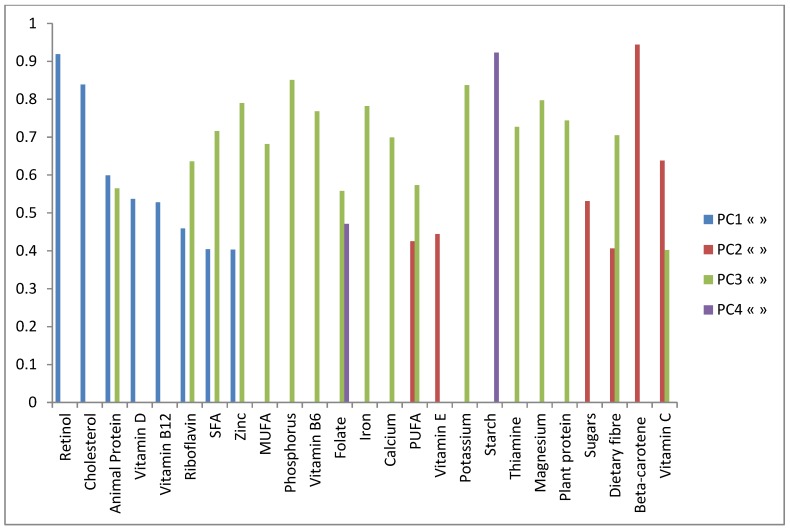
Plotted loadings (all > 0.4) for principal components(1–4).

The number of retained principal components (PC) or “patterns” was determined taking into account several criteria which included the interpretation of the patterns, the percentage of total variance explained and the visual inflections in the scree-plots of eigen-values (Figure 1) [47]. Nutrients with absolute loadings greater or equal to +/−0.40 on a given PC were used to name the retained PC and provide a nutritional interpretation (Figure 2). The loadings represent covariance between the nutrients and the patterns. Nutrients with positive loadings were positively associated with a nutrient pattern while negative loadings were inversely associated. PCA was the most appropriate multivariate reduction technique to apply in this sample as demonstrated statistically by a Kaiser-Meyer-Olkin measure of sampling adequacy of 0.9 and Bartlett’s test of sphericity significant at *p* < 0.001.

Multiple linear regression models were fitted for each of the PC scores on the following socio-demographic and lifestyle characteristics: Sex (by category: males, females), age of adolescent (continuous), BMI (continuous), log of total energy intake (continuous), physical activity (continuous: total minutes to and from school per week), maternal educational level (by category: none, primary school, secondary and higher), Tanner stage (by category: early, mid), marital status of mother (by category: ever in union current, ever in union never, ever in union ended), maternal age (by category: 15–24, 25–34, 35–49, >50 years), maternal SES (by category: lowest tertile (third), middle tertile, highest tertile). Regression coefficients and their standard errors are presented. Partial *R*^2^ were calculated to express the proportion of variance of PC scores explained by each of the measured lifestyle variables. The retained principal components were further divided into tertiles, based on individual PC scores. Analysis of variance (ANOVA) (continuous variables) and chi-squared test (categorical variables) were used to compare differences across tertiles for socio-demographic, anthropometric and dietary intakes.

Multiple linear regression models were computed for each of the PC scores with BMI for age Z scores as an outcome (dependent variable-continuous). Regression coefficients for 1SD increase in PC scores for each depicted nutrient pattern were computed for three models M1: (crude), M2: (adjusted for M1 plus physical activity), M3: (adjusted for M2 plus SES of mother) and M4: (adjusted for M3 plus educational level of the mother). Mutually adjusting for all PCs did not affect the above mentioned models. All statistical significance were defined using a 2-sided *p*-value < 0.05.

## 3. Results

### 3.1. Identification and Description of Depicted Nutrient Patterns (PC)

Four nutrient patterns, which explained about 79% of the total variance (total nutrient variability), were retained by the overall PCA (*N* = 388) (Table 1). The 1st PC retained had largest positive loadings on animal protein, saturated fat, cholesterol, riboflavin, vitamin B12, retinol, vitamin D and zinc (nutrients mainly of animal origin). Because of these positive loadings this PC was named “*Animal driven nutrients*”. This pattern accounted for 26% of the variance in nutrient intakes. The 2nd PC had the greatest positive loadings on the following vitamins: vitamin C, beta-carotene, and vitamin E. Additionally, dietary fibre, PUFA and sugars also had strong positive loadings. Because of these loadings this PC was named “*Vitamins*, *fibre and vegetable oil nutrients*”. This pattern accounted for 21% of the variance in nutrient intakes and is distinctively different from PC1. The 3rd PC was named “*mixed diet driven nutrients*” because of its heterogeneous nature in that both animal and plant derived nutrients had large positive loadings on the matrix. The greatest positive loadings were on the following (i) vitamins and minerals: thiamine, riboflavin, vitamin B12, vitamin B6, folate, vitamin C, calcium, phosphorus, iron, potassium, magnesium and zinc and; (ii) other nutrients: animal protein, plant protein, saturated fat, MUFA, PUFA and dietary fibre. This pattern accounted for 19% of the variance in nutrient intakes. The 4th and last PC retained accounted for 13% of the variance in nutrient intakes. This PC had the largest loadings on starch and folate and was termed the “*Starch and folate driven pattern*”.

**Table 1:** Principal Components (PC) loading matrix and explained variances for the first four nutrient patterns identified by PCA in rural black South African adolescents: Agincourt.

Nutrients	PC1 (Animal Driven Nutrients)	PC2 (Vitamins, Fibre and Vegetable Oil Nutrients)	PC3 (Mixed Diet Driven Nutrients)	PC4 (Starch and Folate Driven)
Animal Protein	0.599	−0.032	0.565	−0.019
Plant protein	0.127	0.255	0.744	0.249
SFA	0.404	0.205	0.716	0.179
MUFA	0.356	0.326	0.682	0.191
PUFA	0.298	0.425	0.573	0.25
Cholesterol	0.839	−0.005	0.371	0.115
Starch	0.207	0.235	0.165	0.923
Sugars	0.12	0.531	0.276	0.364
Dietary fibre	0.115	0.406	0.705	0.152
Thiamine	0.184	0.053	0.727	0.002
Riboflavin	0.459	0.105	0.636	0.058
Vitamin B_6_	0.344	0.261	0.768	0.174
Folate	0.316	0.329	0.558	0.471
Vitamin B_12_	0.528	0.059	0.27	0.089
Vitamin C	0.04	0.638	0.402	0.089
Beta-carotene	0.083	0.944	0.136	0.081
Retinol	0.919	0.211	-0.007	0.157
Vitamin E	0.243	0.444	0.381	0.277
Vitamin D	0.537	0.205	0.359	0.148
Calcium	0.307	0.33	0.699	0.118
Phosphorus	0.349	0.163	0.851	0.092
Iron	0.31	0.253	0.782	0.116
Potassium	0.239	0.323	0.837	0.172
Magnesium	0.128	0.308	0.797	0.043
Zinc	0.403	0.172	0.79	0.129
Explained variance (%)	26	21	19	13
Cumulative explained variance (%)	26	47	66	79

Principle Component Analysis (PCA) on 25 log-transformed nutrients adjusted for total energy intake (equivalent to alcohol-free energy in this adolescent sample). PCA, Saturated Fatty Acids (SFA), Monounsaturated Fatty Acids (MUFA), Polyunsaturated Fatty Acids (PUFA). Variance based on rotated Sums of Squared Loadings; Rotation method: Varimax with Kaiser Normalisation.

### 3.2. Dietary, Lifestyle, Anthropometric and Socio-Demographic Variables Associated with Identified Nutrient Patterns

Table 2 and Table 3 show regression coefficients and partial R-squared of individual PC scores for each of the four patterns retained for energy, anthropometric, lifestyle and socio-demographic variables. Energy intake (log) was positively and significantly associated with all four patterns (*p* < 0.0001). Being female, never in union as marital status of mother of the adolescent, and maternal age between 35 and 49 years were positively and significantly associated with PC1 “*Animal driven nutrients*” (*p* ≤ 0.05), whilst being in the lowest SES status tertile was negatively associated with the same PC (*p* ≤ 0.05). Being in mid-puberty was positively and significantly associated with PC2 “*Vitamins, fibre and vegetable oil nutrients*” (*p* ≤ 0.05). Physical activity (walking to and from school) and being in the lowest SES status tertile were positively associated with PC3 “*mixed diet driven nutrients*” (*p* ≤ 0.05). PC4 was negatively associated with physical activity (walking to and from school) (*p* ≤ 0.05) (Table 2). Variability explained by socio-demographic, anthropometric, lifestyle factors and energy intake for the four PCs is presented in Table 3. Energy intake significantly explained the most variability with 6.5%, 6%, 66.6%, and 6.7% for PC1, PC2, PC3 and PC4 respectively (*p* < 0.0001). Sex (2.6%: *p* < 0.0001), maternal education (5.9%: *p* < 0.001), BMI (1.3%: *p* ≤ 0.03) and SES of mother (1.3%: *p* ≤ 0.04) significantly contributed to the variability in PC1 “*Animal driven nutrients*”. Tanner stage significantly contributed to the variability (1.1%: *p* ≤ 0.04) in PC2 “*Vitamins*, *fibre and vegetable oil nutrients*”, whilst physical activity (minutes walking to and from school per week) significantly contributed to the variability (1.3%: *p* ≤ 0.04) in PC4 “*Starch and folate driven pattern*”.

**Table 2:** Multiple regression derived coefficients (β) and standard errors (SE) of specified predictors for the four nutrient pattern scores.

Variables	PC1 (Animal Driven Nutrients)			PC2 (Vitamins, Fibre and Vegetable Oil Nutrients)			PC3 (Mixed Diet Driven Nutrients)			PC4 (Starch and Folate Driven)		
	β	SE	*p*-Value	β	SE	*p*-Value	β	SE	*p*-Value	β	SE	*p*-Value
Age (years) of adolescent	−0.027	0.036	0.46	−0.046	0.036	0.21	0.033	0.022	0.14	0.007	0.037	0.84
BMI	0.024	0.016	0.15	−0.009	0.016	0.58	−0.005	0.1	0.64	0.001	0.017	0.98
Log (Energy)	0.607	0.132	<0.0001	0.601	0.132	<0.0001	2.127	0.08	<0.0001	0.665	0.135	<0.0001
Sex												
Female	0.235	0.11	0.03	−0.052	0.11	0.64	−0.046	0.067	0.5	0.147	0.112	0.19
Male (Ref)												
Maternal Education level												
Primary school	−0.041	0.13	0.75	0.09	0.131	0.49	−0.07	0.079	0.38	−0.16	0.133	0.24
Secondary and higher education	−0.147	0.138	0.29	0.171	0.138	0.22	−0.109	0.084	0.2	−0.57	0.141	0.69
None (Ref)												
Physical activity of adolescent												
Walking to and from school (min week^−1^)	<0.0001	<0.0001	0.62	<0.0001	<0.0001	0.74	0.001	0.001	0.01	−0.001	0.001	0.05
Tanner stage												
Mid	−0.067	0.151	0.66	0.343	0.151	0.02	−0.045	0.092	0.62	0.014	0.154	0.93
Early (Ref)												
Marital Status of mother												
Never in union	0.231	0.121	0.05	0.196	0.121	0.11	−0.108	0.703	0.14	0.106	0.124	0.4
Ever in union ended	0.017	0.183	0.93	0.104	0.183	0.57	0.051	0.11	0.65	0.001	0.187	0.99
Ever in union current (Ref)												
Maternal age (years)												
15–24	0.289	0.975	0.77	0.966	0.978	0.32	−0.257	0.59	0.66	0.406	0.996	0.68
25–34 (Ref)												
35–49	0.264	0.115	0.02	0.007	0.115	0.95	−0.09	0.07	0.2	0.048	0.117	0.68
>50	−0.251	0.178	0.161	0.188	0.179	0.29	0.117	0.108	0.28	0.076	0.182	0.68
SES status of mother (based on SES Wealth Index)												
lowest tertile	−0.336	0.134	0.01	−0.098	0.134	0.46	0.166	0.081	0.04	−0.06	0.136	0.67
middle tertile	−0.11	0.132	0.4	−0.207	0.133	0.12	0.071	0.08	0.39	−0.07	0.135	0.62
highest tertile (Ref)												

PC scores had means of 0 but standardized to unit variance; PC scores calculated from QFF derived intake of 25 nutrients, *n* = 388; SES, socio-economic status socio-economic status; BMI, body mass index; Ref, reference group in the regression after creating dummy variables (categorical coding in regression analysis).

**Table 3:** *p*-Values of *F*-test on type III sum of squares estimate.

Variable	DF#	PC1 (Animal Driven Nutrients)			PC2 (Vitamins, Fibre and Vegetable Oil Nutrients)			PC3 (Mixed Diet Driven Nutrients)			PC4 (Starch and Folate Driven)		
		Partial *R*^2^	%	*p*-value	Partial *R*^2^	%	*p*-value	Partial *R*^2^	%	*p*-value	Partial *R*^2^	%	*p*-value
Age	1	0.001	0.1	0.57	0.001	0.1	0.48	0.009	0.9	0.06	0.001	0.1	0.51
BMI	1	0.013	1.3	0.03	0.001	0.1	0.64	0.003	0.3	0.28	0.006	0.6	0.12
Log(Energy)	1	0.065	6.5	<0.0001	0.06	6	<0.0001	0.666	66.6	<0.0001	0.067	6.7	<0.0001
Sex	1	0.026	2.6	<0.0001	0	0	0.85	0.005	0.5	0.17	0.004	0.4	0.23
Maternal education	2	0.059	5.9	<0.0001	0.006	0.6	0.2	0.002	0.2	0.51	0	0	0.84
Physical activity (Walking to and from school (min week^−1^)	1	0.005	0.5	0.17	0.001	0.1	0.5	0	0	0.96	0.013	1.3	0.02
Tanner Stage	1	0.001	0.1	0.65	0.011	1.1	0.04	0.002	0.2	0.33	0.002	0.2	0.41
Marital status of mother	2	0.001	0.1	0.51	0.008	0.8	0.1	0.001	0.1	0.57	0.001	0.1	0.61
Maternal age	3	0.002	0.2	0.4	0.001	0.1	0.55	0.001	0.1	0.59	0.001	0.1	0.6
SES status of mother (based on SES Wealth Index)	2	0.013	1.3	0.04	0.01	1	0.07	0	0	0.84	0.005	0.5	0.23

PC scores had means of 0 but standardized to unit variance; PC scores calculated from QFF derived intake of 25 nutrients, *n* = 388, # degrees of freedom; socio-economic status socio-economic status.

**Table 4:** Regression coefficients for BMI for Age Z scores for 1 SD increase in PC 1 (*Animal driven nutrients*) and PC4 (Starch and folate driven) scores in rural black South African adolescents.

Nutrient pattern	M1		M2		M3		M4	
	B (95% CI)	*p* value	B (95% CI)	*p* value	B (95% CI)	*p* value	B (95% CI)	*p* value
**PCI**	0.129 (0.018–0.239)	0.02	0.137 (0.025; 0.248)	0.02	0.118 (0.005; 0.230)	0.04	0.120 (−0.005; 0.245)	0.06
Physical activity			2.191×10^−5^ (−0.001; 0.001)	0.95	0.0 (−0.001; 0.001)	0.78	6.67×10^−5^ (−0.001; 0.001)	0.87
**Socio-economic status**								
low tertile					−0.331 (−0.601; −0.060)	0.02	−0.346 (−0.672; −0.020)	0.04
middle tertile					0.009 (−0.262; 0.280)	0.95	0.144 (−0.172; 0.46)	0.37
highest tertile (ref)								
**Maternal education**								
Total number of years of schooling							0.026 (0.0; 0.053)	0.05
**PC4**	0.103 (−0.008; 0.213)	0.05	0.092 (−0.022; 0.206)	0.12	0.087 (−0.026; 0.201)	0.13	0.011 (−0.117;0.138)	0.87
Physical activity			2.74× 10^−5^ (−0.001; 0.001)	0.94	0.0 (−0.001; 0.001)	0.75	7.59×10^−6^ (−0.001; 0.001)	0.99
**Socio-economic status**								
low tertile					−0.361 (−0.629; −0.092)	0.01	−0.407 (−0.729; −0.085)	0.01
middle tertile					0.002 (−0.269; 0.273)	0.98	0.103 (−0.212; 0.418)	0.52
highest tertile (ref)								
**Maternal education**								
Total number of years of schooling							0.028 (0.002; 0.05)	0.04
**R2 values of each model**	**0.009**		**0.007**		**0.029**		**0.063**	

M1: (crude); M2: (adjusted for physical activity); M3: (adjusted for M2 plus socio-economic status of mother); M4: (adjusted for M3 plus educational level of mother); M1, model 1; M2, model 2; M3, model 3; M4 model 4. Ref, reference group in the regression after creating dummy variables (categorical coding in regression analysis).

Differences across tertiles for each retained PC for dietary, anthropometric, lifestyle and socio-demographic factors are presented in supplementary Table 1. BMI for age Z scores were used as a proxy or indicator of obesity status. Table 4 presents the adjusted increase in BMI for age Z scores per 1 SD increase in each retained PC score. For PC1, a positive significant association was observed with BMI for age Z scores in M1 (0.13 (0.02; 0.24); *p* = 0.02), M2 (0.14 (0.03; 0.25); *p* = 0.02) and M3 (0.12 (0.01; 0.24); *p* = 0.04). Comparable results were observed for PC4 in that a positive association was observed as well with BMI for age Z scores for M1 (0.10 (−0.01; 0.21); *p* = 0.05). No significant associations with BMI for age Z scores were observed for PC2 and PC3 and thus not presented.

## 4. Discussion

This is the first study to our knowledge to identify and describe nutrient patterns and how they relate to various variables/outcomes including obesity in rural black South African adolescents. The PCA technique used for the present analysis has several advantages especially in comparison to the generic factor analysis. PCs retained are generated sequentially, meaning the variance explained by the first factor is removed, and the second factor is then generated to maximally explain the remaining variance in the matrix (this is continuous with successive components [3,4,49]. The definition of each component is independent of the number of components retained [3]. This is useful in identifying various combinations of nutrients that could reflect possible biological mechanisms especially in association with various other health outcomes. Limitations related to PCA include the subjective decisions on how to interpret and name patterns, choice of variables to include in the matrix, whether to transform or standardize data, the number of components to retain, and the threshold for factor loadings to be used in naming patterns (*i.e.*, |+/−0.40| in this analysis) [47]. Furthermore, PCA derived patterns can be used as a standard approach to describe dietary habits of populations but the use of these patterns in examining diet–disease relationships has a minor limitation in that although PCA aims to maximize the fraction of variance explained by a weight linear combination of original variables, this however does not necessarily increase the ability to discriminate between subjects with disease or not.

We identified four nutrient patterns explaining 79% of the total variance in nutrient intakes: PCI (26%) was characterized by animal derived nutrients; PC2 (21%) was characterized by *vitamins, fibre and vegetable oils*; PC3 (19%) was characterized by both animal and plant derived nutrients (*mixed diet driven nutrients)* and; PC4 (13%) was characterized by starch and folate. All studies published so far on nutrient patterns have been conducted in non-African regions and populations. PCI is consistent and comparable to patterns depicted in previous studies labelled as “meat” [14,15], “high meat” [12,21], “animal products” [8,9,50,51] which were similarly characterized by high positive loadings of nutrients from animal derived sources. This additionally illustrates the adoption of westernized diets as PC1 explains most of the variance in this rural setting. PC2 which had high loadings for vitamins, fibre and vegetable oils nutrients is comparable to patterns reported in previous studies labelled as “fibre and vitamins” [8,9,10,11,16,22,50,51], “vitamin rich” [20], and “antioxidant vitamins and fibre” [17]. PC3 had a high heterogeneous contribution of both animal and plant derived nutrients, characterizing both a “Mediterranean nutrient pattern” and a “Western-like pattern”. This two-in-one combination has been shown elsewhere [2,52]. PC4 is unique to this study and has not been reported elsewhere.

With PC1 and PC4, a positive and significant association was observed with BMI for age Z scores whilst no significant associations were observed for either PC2 or PC3. Observed positive associations between PCI “animal driven nutrients” and BMI for age Z scores are consistent and comparable to those observed for western driven patterns reported in the literature [53,54,55,56]. Associations observed for PC4 with BMI for age Z scores are the first to be reported in the present study and are attributed to high starch and folate loadings. It should be noted that PC4 was not associated with BMI for age Z scores after full adjustment. In this rural setting, these associations could be explained in that we seem to be observing at household level that improved education status is positively driving household SES. Rural households’ diets differ with changing SES; improved SES is characterised by less physical activity and increased obesity risk (Table 4).

Though to the best of our knowledge, the food list of the QFFQ used to assess usual diet in this study is known to be comprehensive as reported elsewhere [38,39,40], we cannot ignore the inherent limitations around measurement errors and the complexity of assessing dietary intake in all nutritional epidemiological studies using self-reported diet. The use of dietary supplements was not included in the calculations of nutrient pattern scores, though these were unlikely to be used to any extent by adolescents in rural South African settings. Energy intake as expected was the most important factor explaining variability in PC scores, despite adjusting for it in the present analysis. Normalization for total energy helps to remove variation due to body size and metabolic rates and contributes to reducing measurement error in reported dietary intakes. Due to the cross sectional nature of the data presented here, the associations observed between the retained PCs and socio-demographic, lifestyle factors, and obesity risk cannot infer causality (problem of reverse causation should be noted) thus longitudinal data are required to do this.

Compared with food patterns, studying nutrient patterns have several advantages including that nutrients are universal, functionally not exchangeable, and in contrast to food patterns may characterize specific nutritional profiles in an easier way for comparison to other populations. Additionally, nutrients, unlike foods, show a limited number of non-consumers, and this approach could better mirror a combination of bioactive nutrients in complex biological mechanisms associated with diseases and obesity as compared to food patterns. From a public health perspective, since, in contrast to foods, nutrients are universal, this allows and supports the development or adaptation of existing food based dietary guidelines (FBDGs) using a variety of different interchangeable foods and/or food groups that mimic each other in nutrients. Different foods can have the same nutrient densities yet they are not equally and easily accessible to all geographic regions in a country (substitution with a food containing more or less the same nutrients should be stressed in FDBGs).

## 5. Conclusions

The present analysis confirmed a large variability in nutrient intakes but we were able to retain four nutrient patterns that were related to various socio-demographic and lifestyle factors, including BMI. Both poorer households and those with improving socio-economic status are placing adolescents at risk of obesity given the concomitant nutrient patterns and lifestyle behaviors. It is critical that intervention programs constructively address the consequences of the economic and nutrition transition underway in South Africa by assisting healthier diet choices around reduced carbohydrate intake, increasing food diversity, and promoting active lifestyles.

## Supplementary Files

Supplementary File 1
