# Supplementary material for: Nutrient Patterns and Their Association with Socio-Demographic, Lifestyle Factors and Obesity Risk in Rural South African Adolescents
Source: Nutrients. 2015 May 12;7(5):3464–82. doi: 10.3390/nu7053464 (PMC4446762; doi:10.3390/nu7053464)
Supplement: Supplementary File 1 [file nutrients-07-03464-s001.docx]

**Supplementary Information**

**Table S1.** Socio-demographic, anthropometric parameters and dietary intakes of the study population according to tertiles for each of the four depicted nutrient pattern scores (*n* = 388).

|  | **PC1** | | | **PC2** | | | **PC3** | | | **PC4** | | |
| --- | --- | --- | --- | --- | --- | --- | --- | --- | --- | --- | --- | --- |
|  | **T1** | **T2** | **T3** | **T1** | **T2** | **T3** | **T1** | **T2** | **T3** | **T1** | **T2** | **T3** |
| Age (year)^1^ | 13.6 ± 1.6 | 13.4 ± 1.7 | 13.7 ± 1.6 | 13.6 ± 1.6 | 13.6 ± 1.7 | 13.5 ± 1.6 | 13.4 ± 1.6 | 13.5 ± 1.7 | 13.8 ± 1.6 | 13.5 ± 1.6 | 13.6 ± 1.7 | 13.6 ± 1.5 |
| Girls (%)^2^ | 39 | 55 | 58* | 48 | 47 | 54 | 53 | 49 | 48 | 42 | 62 | 47* |
| BMI at baseline (kg m^−2^) ^1^ | 18.6 ± 3.3 | 18.5 ± 3.1 | 19.6 ± 4.3* | 18.8 ± 3.4 | 18.8 ± 3.4 | 19.0 ± 3.9 | 18.5 ± 2.9 | 19.3 ± 4.4 | 19.0 ± 3.3 | 18.5 ± 3.4 | 18.8 ± 3.2 | 19.3 ± 3.6 |
| BMI for Age Z scores ^1^ | −0.3 ± 1.0 | −0.4 ± 1.1 | −0.1 ± 1.2* | −0.3 ± 1.1 | −0.3 ± 1.1 | −0.2 ± 1.2 | −0.3 ± 1.0 | −0.2 ± 1.2 | −0.3 ± 1.1 | −0.4 ± 1.1 | −0.3 ± 1.1 | −0.1 ± 1.1 |
| Waist to height ratio ^1^ | 0.4 ± 0.0 | 0.4 ± 0.1 | 0.4 ± 0.1 | 0.4 ± 0.1 | 0.4 ± 0.1 | 0.4 ± 0.0 | 0.4 ± 0.0 | 0.4 ± 0.1 | 0.4 ± 0.0 | 0.4 ± 0.0 | 0.4 ± 0.0 | 0.4 ± 0.1 |
| Waist to hip ratio ^1^ | 0.9 ± 0.6 | 0.8 ± 0.1 | 0.9 ± 0.9 | 0.9 ± 0.9 | 0.9 ± 0.7 | 0.8 ± 0.1 | 0.9 ± 0.9 | 0.8 ± 0.7 | 0.8 ± 0.1 | 0.9 ± 0.6 | 0.8 ± 0.1 | 0.9 ± 0.9 |
| Maternal education status (secondary and higher) (%) ^2^ | 34 | 25 | 22* | 24 | 28 | 28 | 20 | 32 | 23 | 34 | 29 | 18 |
| Physical activity (mins walked to and from school per week) ^1^ | 200.0 ± 137.8 | 201.0 ± 156.2 | 194.0 ± 150.2 | 218.3 ± 170.6 | 181.8 ± 119.5 | 202.9 ± 165.2 | 206.2 ± 165.0 | 196.3 ± 137.2 | 202.7 ± 159.5 | 220.3 ± 147.5 | 215.6 ± 162.7 | 173.7 ± 144.7# |
| SES status of mother (lowest SES tertile) (%) ^2^ | 41 | 33 | 30 | 37 | 30 | 38 | 31 | 35 | 36 | 34 | 35 | 33 |
| Tanner Stage (Early) (%) ^2^ | 16 | 19 | 17 | 23 | 17 | 14 | 19 | 22 | 12 | 21 | 15 | 17 |
| Maternal age (25-34 years) (%)^2^ | 35 | 31 | 37 | 33 | 36 | 35 | 32 | 33 | 37 | 34 | 33 | 37 |
| Marital status of mother (ever in union, current) (%) ^2^ | 61 | 53 | 51 | 55 | 52 | 51 | 52 | 56 | 51 | 57 | 51 | 53 |
| Total energy intake (kilojoules day^−1^) ^1^ | 6053.7 ± 2205.3 | 6991.6 ± 2606.8 | 7741.9 ± 2976.9# | 6096.4 ± 2197.6 | 6935.5 ± 2106.1 | 7785.1 ± 3192.5# | 4858.1 ± 1290.8 | 6783.2 ± 1432.2 | 9328.6 ± 2733.4# | 5815.5 ± 2271.8 | 7177.6 ± 2610.6 | 7664.2.6 ± 2836.6# |
| Total protein (g day^−1^) ^1,3^ | 35.4 ± 13.7 | 44.4 ± 18.7 | 56.4 ± 33.0# | 40.5 ± 16.3 | 45.1 ± 17.2 | 48.2 ± 23.4* | 29.8 ± 11.1 | 43.3 ± 11.3 | 63.8 ± 31.4 | 40.9 ± 32.9 | 46.8 ± 20.2 | 47.2 ± 20.0 |
| Animal protein (g day^−1^) ^1,3^ | 8.3 ± 6.5 | 14.9 ± 9.8 | 25.4 ± 29.2# | 14.3 ± 9.7 | 16.0 ± 11.8 | 15.4 ± 14.0* | 9.2 ± 8.9 | 14.1 ± 8.9 | 25.3 ± 29.2 | 16.0 ± 30.2 | 17.0 ± 12.5 | 14.6 ± 11.1# |
| Plant protein (g day^−1^) ^1,3^ | 27.0 ± 10.4 | 29.4 ± 12.0 | 30.4 ± 12.1# | 26.1 ± 10.1 | 28.8 ± 9.5 | 32.4 ± 13.6 | 20.4 ± 5.8 | 28.9 ± 7.1 | 38.2 ± 12.1* | 24.9 ± 8.7 | 29.4 ± 11.8 | 32.3 ± 12.4# |

**Table S1. *Cont.***

| Carbohydrates (g day−1) ^1,3^ | 235.5 ± 83.2 | 257.8 ± 98.0 | 264.7 ± 98.1# | 232.0 ± 80.2 | 253.6 ± 76.8 | 276.7 ± 113.0# | 190.1 ± 57.0 | 247.1 ± 54.3 | 326.3 ± 99.9 | 216.4 ± 70.8 | 253.6 ± 93.5 | 282.5 ± 96.4# |
| --- | --- | --- | --- | --- | --- | --- | --- | --- | --- | --- | --- | --- |
| Starch (g day^−1^) ^1,3^ | 18.8 ± 26.3 | 23.9 ± 32.2 | 33.6 ± 41.9# | 26.6 ± 37.0 | 27.9 ± 32.5 | 27.1 ± 34.6 | 17.5 ± 22.4 | 26.5 ± 32.5 | 32.7 ± 43.9# | 2.1 ± 2.5 | 15.4 ± 9.4 | 59.0 ± 41.2# |
| Total Fat (g day^−1^) ^1,3^ | 30.2 ± 16.8 | 40.3 ± 22.1 | 51.5 ± 28.0# | 31.6 ± 19.5 | 40.9 ± 18.7 | 49.0 ± 28.0# | 23.6 ± 11.0 | 40.2 ± 16.3 | 59.6 ± 27.9 | 30.8 ± 22.7 | 45.7 ± 23.1 | 45.2 ± 26.1# |
| Saturated Fat (g day^−1^) ^1,3^ | 7.4 ± 4.6 | 10.1 ± 6.0 | 13.8 ± 9.8# | 8.4 ± 5.5 | 10.3 ± 5.7 | 11.8 ± 7.7 | 5.6 ± 3.0 | 10.2 ± 5.2 | 15.6 ± 9.4 | 8.0 ± 8.7 | 11.7 ± 6.4 | 11.2 ± 7.5* |
| MUFA (g day^−1^) ^1,3^ | 10.5 ± 6.6 | 14.0 ± 8.5 | 17.3 ± 10.3# | 10.4 ± 6.9 | 14.1 ± 7.1 | 16.6 ± 9.8# | 7.7 ± 4.0 | 13.4 ± 6.0 | 20.7 ± 10.4* | 10.7 ± 9.1 | 15.5 ± 8.4 | 15.1 ± 9.1* |
| PUFA (g day^−1^) ^1,3^ | 9.4 ± 6.0 | 12.6 ± 8.5 | 15.3 ± 9.0# | 9.5 ± 7.7 | 12.6 ± 6.2 | 16.0 ± 10.5# | 7.7 ± 4.0 | 12.7 ± 6.3 | 17.8 ± 10.9 | 8.9 ± 5.9 | 14.2 ± 9.1 | 14.6 ± 9.5* |
| Cholesterol (mg day^−1^) ^1,3^ | 18.0 ± 14.4 | 45.4 ± 29.7 | 151.3 ± 128.3# | 61.0 ± 72.1 | 71.9 ± 79.0 | 74.0 ± 110.5 | 43.9 ± 84.4 | 75.3 ± 81.0 | 95.5 ± 114.6# | 59.3 ± 111.9 | 70.3 ± 66.5 | 85 ± 107.0 |
| Fiber (g day^−1^) ^1,3^ | 19.4 ± 9.7 | 21.3 ± 10.8 | 22.0 ± 9.3* | 17.2 ± 6.9 | 20.0 ± 6.6 | 26.2 ± 13.3# | 14.2 ± 4.8 | 20.9 ± 5.5 | 28.4 ± 12.2* | 17.5 ± 6.9 | 22.2 ± 9.2 | 22.6 ± 11.1 |
| Vitamin C (mg day^−1^) ^1,3^ | 85.9 ± 114.4 | 84.6 ± 81.9 | 91.1 ± 93.3 | 57.6 ± 74.4 | 72.7 ± 53.8 | 148.9 ± 212.7# | 52.2 ± 54.6 | 79.2 ± 68.7 | 146.6 ± 215.3* | 67.3. ± 77.3 | 91.6 ± 84.7 | 106.6 ± 205.7 |
| ß-Car. (μg day^−1^) ^1,3^ | 1121.6 ± 1320.0 | 1275.2 ± 1603.5 | 1424.9 ± 1745.7 | 248.0 ± 199.1 | 952.0 ± 461.6 | 2618.7 ± 2002.7# | 1017.1 ± 1295.2 | 1262.3 ± 1443.6 | 1424.9 ± 1860.3* | 1032.3 ± 1444.6 | 1578.9 ± 1835.7 | 1218.2 ± 1339.6* |
| Vitamin D (µg day^−1^) ^1,3^ | 1.0 ± 1.1 | 1.5 ± 1.4 | 3.3 ± 2.5# | 1.4 ± 1.7 | 2.1 ± 2.1 | 2.2 ± 2.2 | 1.0 ± 1.3 | 2.1 ± 2.0 | 2.7 ± 2.5 | 1.4 ± 1.8 | 2.1 ± 2.1 | 2.2 ± 2.2 |
| Retinol (µg day^−1^) ^1,3^ | 1.2 ± 1.7 | 8.1 ± 6.0 | 150.6 ± 542.6# | 21.7 ± 81.0 | 30.1 ± 133.5 | 109.2 ± 529.1 | 109.7 ± 552.4 | 26.7 ± 62.8 | 29.8 ± 153.5# | 72.2 ± 516.7 | 20.3 ± 45.4 | 72.5 ± 239.6 |
| Riboflavin (mg day^−1^) ^1,3^ | 0.5 ± 0.4 | 0.7 ± 0.5 | 1.0 ± 0.7# | 0.6 ± 0.6 | 0.7 ± 0.5 | 0.8 ± 0.7 | 0.4 ± 0.4 | 0.6 ± 0.3 | 1.1 ± 0.8* | 0.6 ± 0.5 | 0.8 ± 0.7 | 0.5 ± 0.8# |
| Thiamine (mg day^−1^) ^1,3^ | 1.0 ± 0.9 | 1.2 ± 1.2 | 1.3 ± 0.8 | 1.2 ± 1.0 | 1.2 ± 0.9 | 1.2 ± 1.0* | 0.7 ± 0.3 | 1.0 ± 0.4 | 2.0 ± 1.3# | 1.2 ± 1.1 | 1.3 ± 1.1 | 1.1 ± 0.7# |
| Folate (µg day^−1^) ^1,3^ | 156.2 ± 83.3 | 178.2 ± 88.8 | 228.1 ± 134.5# | 155.1 ± 84.8 | 189.0 ± 91.0 | 224.0 ± 132.1 | 138.6 ± 103.0 | 188.2 ± 80.7 | 243.2 ± 112.5# | 136.0 ± 95.3 | 176.4 ± 75.5 | 250.5 ± 120.7# |
| Vitamin B12 (µg day^−1^) ^1,3^ | 1.0 ± 1.5 | 1.5 ± 2.0 | 3.8 ± 4.8# | 1.5 ± 2.1 | 2.1 ± 2.9 | 2.3 ± 4.2 | 1.4 ± 3.5 | 1.8 ± 2.1 | 3.0 ± 4.1 | 1.8 ± 4.0 | 1.9 ± 2.8 | 2.3 ± 3.1 |
| Vitamin B6 (mg day^−1^) ^1,3^ | 0.6 ± 0.4 | 0.8 ± 0.5 | 1.0 ± 0.6* | 0.6 ± 0.4 | 0.8 ± 0.4 | 0.9 ± 0.6 | 0.5 ± 0.2 | 0.7 ± 0.3 | 1.2 ± 0.6 | 0.5 ± 0.6 | 0.9 ± 0.5 | 0.8 ± 0.5# |
| Iron (mg day^−1^) ^1,3^ | 7.1 ± 3.2 | 8.4 ± 4.4 | 9.9 ± 5.1 | 7.2 ± 3.9 | 8.3 ± 3.2 | 9.8 ± 5.3 | 5.6 ± 2.4 | 7.8 ± 2.3 | 12.1 ± 5.0* | 7.4 ± 4.0 | 9.1 ± 5.0 | 8.6 ± 4.0# |
| Zinc (mg day^−1^) ^1,3^ | 4.4 ± 1.7 | 5.5 ± 2.3 | 7.0 ± 4.3# | 4.9 ± 2.1 | 5.5 ± 2.2 | 6.2 ± 3.0 | 3.7 ± 1.2 | 5.5 ± 1.6 | 7.8 ± 4.1 | 5.0 ± 4.1 | 6.0 ± 2.7 | 5.9 ± 2.6# |
| Calcium (mg day^−1^) ^1,3^ | 266.0 ± 168.4 | 327.7 ± 229.6 | 389.5 ± 225.2 | 276.4 ± 197.4 | 332.2 ± 201.1 | 373.3 ± 232.8 | 188.6 ± 93.7 | 296.5 ± 116.7 | 497.5 ± 256.6# | 282.0 ± 213.0 | 3357.6 ± 232.6 | 326.7 ± 188.5# |

^1^ Continuous variable: all values are means ± SD (P values based on ANOVA). ^2^ Categorical variables: all values are percentages (*p* values based on chi-square test). ^3^ Dietary intakes compared (ANCOVA) across tertiles after adjusting for total energy intake. * Significant difference based at level *p* < 0.05; # Significant difference based at level *p* < 0.001.
